# FOXO3-Activated circFGFBP1 Inhibits Extracellular Matrix Degradation and Nucleus Pulposus Cell Death via miR-9-5p/BMP2 Axis in Intervertebral Disc Degeneration In Vivo and In Vitro

**DOI:** 10.3390/ph16030473

**Published:** 2023-03-22

**Authors:** Yanlin Tan, Xiaobin Wang, Yi Zhang, Zhehao Dai, Jing Li, Chuning Dong, Xingwang Yao, Chang Lu, Fei Chen

**Affiliations:** 1 Department of Nuclear Medicine, The Second Xiangya Hospital of Central South University, Changsha 410011, China; 2 Department of Spine Surgery, The Second Xiangya Hospital of Central South University, No.139, Renmin Middle Road, Changsha 410011, China; 3 Department of Surgery Room, The Second Xiangya Hospital of Central South University, Changsha 410011, China

**Keywords:** intervertebral disc degeneration, forkhead box protein O3, circRNA fibroblast growth factor binding protein 1, miR-9-5p, bone morphogenetic protein 2

## Abstract

(1) Background: intervertebral disc degeneration (IVDD) defined as the degenerative changes in intervertebral disc is characterized by extracellular matrix (ECM) degradation and death in nucleus pulposus (NP) cells. (2) Methods: The model of IVDD was established in male Sprague Dawley rats using a puncture of a 21-gauge needle at the endplates located in the L4/5 intervertebral disc. Primary NP cells were stimulated by 10 ng/mL IL-1β for 24 h to mimic IVDD impairment in vitro. (3) Results: circFGFBP1 was downregulated in the IVDD samples. circFGFBP1 upregulation inhibited apoptosis and extracellular matrix (ECM) degradation and promoted proliferation in IL-1β-stimulated NP cells. Additionally, circFGFBP1 upregulation mitigated the loss of NP tissue and the destruction of the intervertebral disc structure in vivo during IVDD. FOXO3 could bind to the circFGFBP1 promoter to enhance its expression. circFGFBP1 upregulated BMP2 expression in NP via sponging miR-9-5p. FOXO3 enhanced the protection of circFGFBP1 in IL-1β-stimulated NP cells, whereas a miR-9-5p increase partly reversed the protection. miR-9-5p downregulation contributed to the survival of IL-1β-stimulated NP cells, which was partially reversed by BMP2 silence. (4) Conclusions: FOXO3 could activate the transcription of circFGFBP1 via binding to its promoter, which resulted in the enhancement of BMP2 via sponging miR-9-5p and then inhibited apoptosis and ECM degradation in NP cells during IVDD.

## 1. Introduction

The intervertebral disc is a cartilaginous structure showing an easier potential of degeneration and ageing compared with other human connective tissues [1]. Due to the limited self-renewal potential, intervertebral disc degeneration (IVDD) is an irreversible change and remarkedly associated with pain, particularly lower back pain [2]. The intervertebral disc consists of fibrous annulus, nucleus pulpous and terminal plates. During IVDD, there is degenerative fibrous structure with extracellular matrix (ECM) degradation and death in nucleus pulposus (NP) cells [3]. ECM degradation is characterized by degradation in collagen fibers and proteoglycan loss, which decrease the capability to withstand mechanical stress [4]. NP cells can produce and release the components of ECM to maintain the structural integrity of the intervertebral disc. Additionally, cells regulate their behaviors, including death, proliferation and differentiation, in response to the altered mechanical properties of ECM [3,5]. Thus, biological function changes in NP cells contribute to the occurrence and development of IVDD. Understanding the causes of altered NP cell function would provide novel insights into IVDD therapy.

Bone morphogenetic protein 2 (BMP2) is a member of the bone morphogenetic protein family and plays a key role in IVDD progression. BMP2 functions as the stimulator of NP cells to form the ECM structure and to induce proteoglycan synthesis, which results in intervertebral disc regeneration. A recent report showed that BMP2 could induce intervertebral disc regeneration via modulating the behaviors of NP cells; in this process, BMP2 activated the PI3K/Akt pathway to inhibit inflammation, apoptosis and ECM disorder in impaired NP cells [6]. BMP2 is found to be downregulated in NP samples from patients with IVDD, according to a bioinformatics analysis based on the Gene Expression Omnibus (GEO) [7]. Similarly, we found that the expression of BMP2 was decreased in the NP tissue of animals undergoing IVDD; however, the cause of BMP2 differential expression remains unclear during IVDD. 

Circular RNAs (circRNAs) are a novel group of non-coding RNAs, of which biofunction is involved in proliferation, senescence, motility, death and ECM metabolism in cells during IVDD [8]. Differentially expressed circRNAs modulate apoptosis and ECM degradation in nucleus pulposus cells, thereby contributing to the pathogenesis of IVDD [9,10]. circFGFBP1 (has_circ_0069234) expression is related to changes in NP tissues undergoing IVDD. We found that circFGFBP1 was significantly downregulated in the NP tissues of patients with IVDD, indicating that circFGFBP1 might be involved in the occurrence and development of IVDD. A bioinformatics analysis by Wang et al. determined that circFGFBP1 was significantly downregulated in IVDD and they predicted that differentially expressed circRNAs in IVDD were associated with degradation, apoptosis and oxidative stress in NP cells during IVDD [11]. Therefore, circFGFBP1 serves as the significant factor of IVDD at the molecular level. Importantly, circRNA functions as the regulator of gene expression in epigenetics via sponging microRNAs (miRNAs), connecting with RNA-binding proteins and modulating RNA splices [12]. We predicted that circFGFBP1 could target the BMP2 expression via sponging miR-9-5p (a miRNA located in human chromosome 1). Based on a bioinformatical tool, we found that BMP2 and cirdFGFBP1 had similar binding sites located in miR-9-5p. Thus, we assumed that a circFGFBP1/miR-9-5p/BMP2 axis may exist in IVDD. In the upstream of circFGFBP1, we assumed that forkhead box O3 (FOXO3) (a transcript factor in the previous study [13]), might be related to the expression of circFGFBP1. We previously found that there was a potential site of FOXO3 located in the promoter of circFGFBP1 and that FOXO3 upregulation led to a circFGFBP1 increase in the in vitro model of IVDD induced by IL-1β stimulation, suggesting the potential relationship between FOXO3 and circFGFBP1 in IVDD. 

Thus, we provide the hypothesis that FOXO3 can activate the transcription of circFGFBP1 via binding to the promoter, which results in the enhancement of BMP2 via sponging miR-9-5p and then inhibits apoptosis and ECM degradation in NP cells during IVDD. This paper aims to reveal the role of the mechanism of circFGFBP1 in ECM degradation and NP cell death during IVDD. Our findings will show the novel insight into IVDD pathogenesis based on a circRNA/miRNA/mRNA axis. Our findings can give the molecular targets of treatment, diagnosis and prognosis of IVDD. 

## 2. Results

### 2.1. Ectopic Expression of circFGFBP1 in NP Cells and Tissues of IVDD

**Figure 1:**
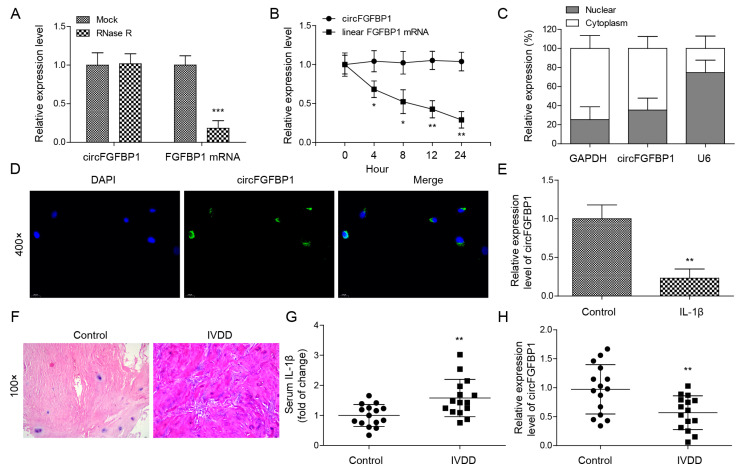
Ectopic expression of circFGFBP1 in NP cells and tissues of IVDD. (**A**) Expressions of circFGFBP1 and FGFBP1 mRNA by qPCR. (**B**) RNA stability assay by actinomycin D. (**C**,**D**) Subcellular distribution of circFGFBP1 by qPCR (**C**) and FISH (**D**). (**E**) CircFGFBP1 expression in NP cells stimulated by IL-1β. *n* = 3. (**F**) Histopathological changes in degenerative intervertebral disc by H&E staining. (**G**) Serum IL-1β by ELISA. (**H**) CircFGFBP1 expression in NP tissues from IVDD patients. *n* = 15. Data were estimated as mean ± SD. * *p* < 0.05, ** *p* < 0.01, *** *p* < 0.001.

We isolated primary NP cells and quantified the circular transcript of circFGFBP1 and its linear mRNA transcript under RNase R treatment. Significantly, we found that the expression of the circular transcript was higher than that of the linear transcript (Figure 1A); furthermore, the circular transcript had better stability compared with the linear one (Figure 1B). Although circFGFBP1 was expressed both in the cytoplasm and the nucleus, circFGFBP1 was mainly distributed in the cytoplasm (Figure 1C,D). We determined that circFGFBP1 was downregulated in IL-1β-stimulated NP cells, mimicking IVDD in vitro (Figure 1E). In addition, we collected NP tissues to determine the histopathological changes during IVDD based on H&E staining. There was a disc volume consisting of oval NP cells in the normal tissues; the disc volume contained abundant glycosaminoglycans (Figure 1F). However, in the NP tissues of IVDD, the disc structure collapsed and fibrous tissues infiltrated the normal tissues (Figure 1F). We determined that the serum IL-1β level was increased in the IVDD group (Figure 1G). Moreover, the expression of circFGFBP1 was decreased in NP tissues from IVDD patients (Figure 1H). Taken together, our results showed that circFGFBP1 is a stable circRNA and may play a key role in IVDD progression.

### 2.2. The Protection of FOXO3 in NP Cell Apoptosis and ECM Degradation via Activating circFGFBP1 Transcription by Binding to Its Promoter Region

**Figure 2:**
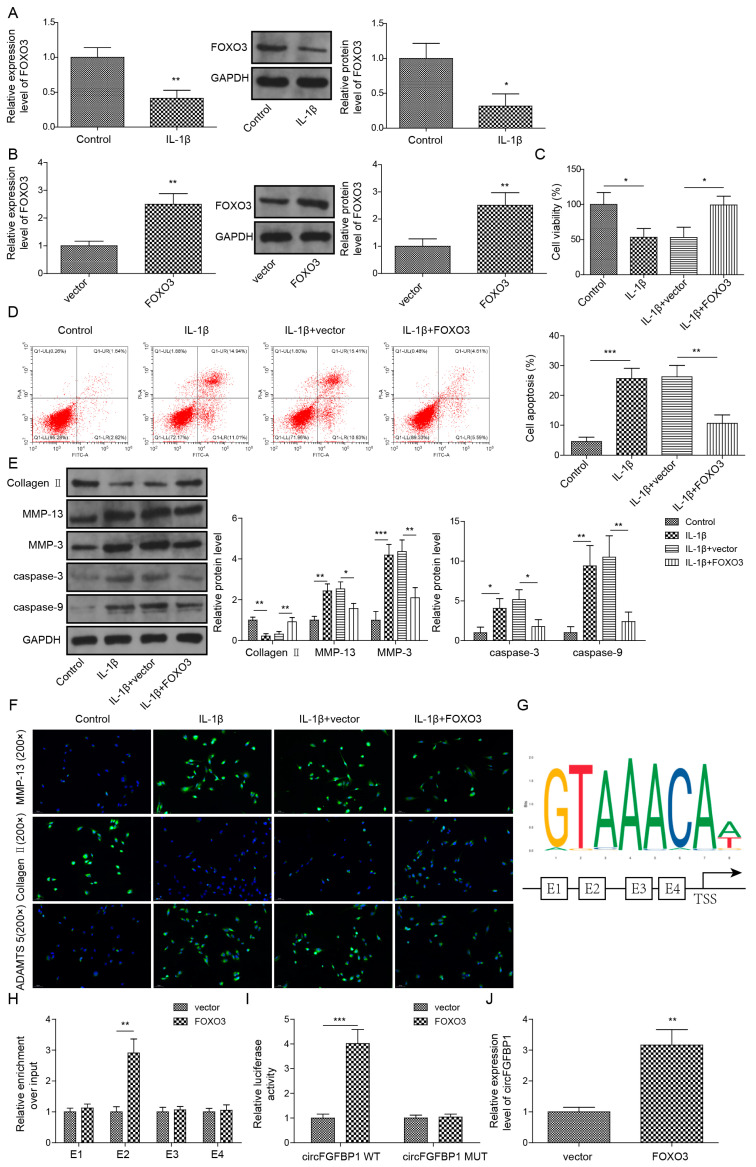
FOXO3 exerts its protection role in NP cell apoptosis and ECM degradation via activating circFGFBP1 transcription by binding to its promoter region. (**A**) Expression of FOXO3 in IL-1β-stimulated NP cells by qPCR and Western blot. (**B**) FOXO3 expression in NP cells transfected with FOXO3 overexpression vector. (**C**) NP cell viability by CCK-8 assay. (**D**) Apoptosis in NP cells by flow cytometry. (**E**,**F**) Expression levels of ECM proteins by Western blot (**E**) and immunofluorescence (**F**). (**G**) The potential site of FOXO3 located in circFGFBP1 promoter by JASPAR. (**H**,**I**) The functional relation between FOXO3 and circFGFBP1 through ChIP (**H**) and dual luciferase reporter gene assay (**I**). (**J**) CircFGFBP1 expression by qPCR under FOXO3 upregulation. Data were estimated as mean ± SD. * *p* < 0.05, ** *p* < 0.01, *** *p* < 0.001.

To construct the cell model of IVDD, we used 10 ng/mL IL-1β to stimulate primary NP cells. Intriguingly, we observed the significant decrease of FOXO3 in IL-1β-stimulated cells (Figure 2A). We transfected the overexpression vector of FOXO3 to upregulate this gene in IL-1β-stimulated cells (Figure 2B). Then, we measured the cell viability of these cells based on CCK-8. Obviously, IL-1β induced the decrease of cell viability in NP cells (Figure 2C). FOXO3 upregulation protected the cell viability from IL-1β stimulation (Figure 2C). Additionally, FOXO3 overexpression could alone increase that in NP cells (Figure S1A). For cell death, FOXO3 decreased IL-1β-increased apoptosis in NP cells (Figure 2D). At the protein level, we found Collagen II was downregulated whereas MMP-13, MMP-3, cleaved Caspase-3 and cleaved Caspase-9 were upregulated in IL-1β-stimulated cells (Figure 2E). Reversely, FOXO3 upregulation partly offset IL-1β-induced changes in theses protein levels (Figure 2E). Further, we observed expressions of Aggrecan, MMP-13 and ADAMTS in NP cells based on the immunofluorescence technique. IL-1β stimulation resulted in the reduction of Aggrecan and the elevations of MMP-13 and ADAMTS5 in NP cells. Due to FOXO3 upregulation, the levels of Aggrecan, MMP-13 and ADAMTS5 in cells stimulated by IL-1β were close to those in normal cells (Figure 2F). Using the JASPAR database, we predicted four potential sites (E1, E2, E3 and E4) of FOXO3 located in the promoter of circFGFBP1 (Figure 2G). We determined that E4 might function as the binding site between FOXO3 and circFGFBP1, according to a ChIP assay (Figure 2H). The site mutation was performed in the E4 region to determine whether FOXO3 could target circFGFBP1 expression via E4. According to the dual luciferase reporter gene assay, we observed that the relative luciferase activity was decreased due to the co-transfection of the FOXO3 overexpression vector and the circFGFBP1 wild type without site mutation rather than the circFGFBP1 mutant (Figure 2I). Moreover, FOXO3 upregulation caused the elevation of circFGFBP1 (Figure 2J). Overall, FOXO3 triggered circFGFBP1 transcription and protected NP cells from IL-1β-induced injury.

### 2.3. Overexpression of circFGFBP1 Prevents Apoptosis and ECM Degradation in NP Cells

**Figure 3:**
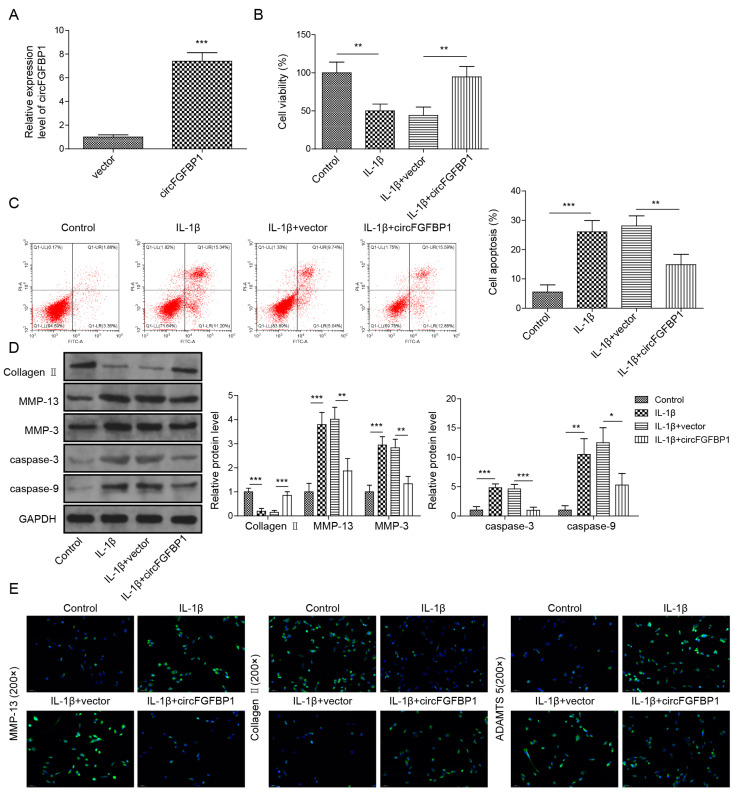
Overexpression of circFGFBP1 prevents apoptosis and ECM degradation in NP cells. (**A**) CircFGFBP1 expression by qPCR. (**B**) Cell viability by CCK-8 assay. (**C**) Apoptosis in NP cells by flow cytometry. (**D**,**E**) Expressions of ECM proteins by Western blot (**E**) and immunofluorescence (**E**). Data were estimated as mean ± SD. * *p* < 0.05, ** *p* < 0.01, *** *p* < 0.001.

We used the overexpression vector of circFGFBP1 to increase this circRNA in NP cells (Figure 3A). Owing to the circFGFBP1 increase, levels of cell viability (Figure 3B) and apoptosis (Figure 3C) in IL-1β-stimulated cells were close to those in normal cells. Cell viability was increased in response to circFGFBP1 upregulation without IL-1β stimulation (Figure S1B). Similar to the role of FOXO3 upregulation, a circFGFBP1 increase induced the enhancement of Collagen II and the reductions of MMP-13, MMP-3, cleaved Caspase-3 and cleaved Caspase-9 to resist the effects of IL-1β stimulation in NP cells (Figure 3D). Additionally, a circFGFBP1 increase partially reversed the regulatory role of IL-1β in protein levels by upregulating Aggrecan and downregulating MMP-13 and ADAMTS5 (Figure 3E). Therefore, circFGFBP1 plays a protective role in IL-1β-induced NP cell injury.

### 2.4. CircFGFBP1 Serves as a miRNA Sponge of miR-9-5p

**Figure 4:**
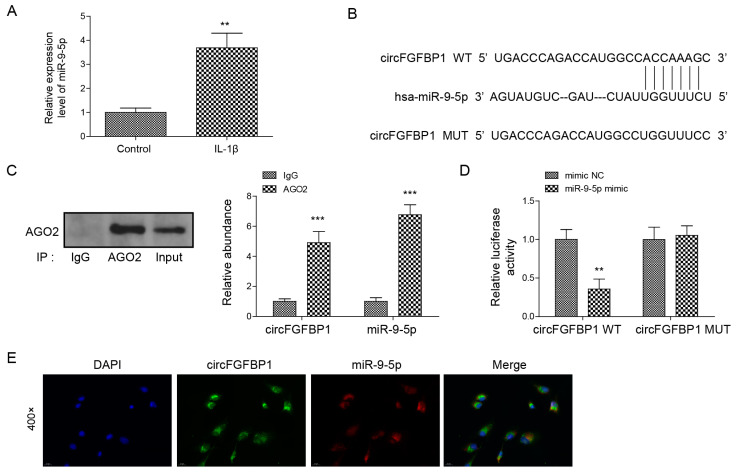
CircFGFBP1 serves as a miRNA sponge of miR-9-5p. (**A**) miR-9-5p expression by qPCR. (**B**) The potential site of circFGFBP1 located in miR-9-5p by starBase. (**C**,**D**) The relationship between circFGFBP1 and miR-9-5p via RIP-qPCR (**C**) and dual luciferase reporter gene assay (**D**). (**E**) The colocalization of circFGFBP1 and miR-9-5p by FISH. Data were estimated as mean ± SD. ** *p* < 0.01, *** *p* < 0.001.

miR-9-5p was upregulated when NP cells underwent IL-1β stimulation (Figure 4A), suggesting the significant role of miR-9-5p in IVDD. Thus, we wondered about the role of miR-9-5p in IVDD. We found that there was a possible binding site of circFGFBP1 located in miR-9-5p (Figure 4B). AGO2 is a miRNA-binding protein. In an RIP assay, we used an anti-AOG2 antibody to precipitate miRNA. If circFGFBP1 was capable of binding to miR-9-5p, circFGFBP1 could be precipitated together due to the immunoprecipitation between AGO2 and miR-9-5p. We observed that the anti-AGO2 antibody precipitated AGO2 and that miR-9-5p was enriched in anti-AGO2 antibody (Figure 4C). Importantly, we found circFGFBP1 enrichment in the anti-AGO2-antibody (Figure 4C), suggesting circFGFBP1 could bind to miR-9-5p. We further wondered whether circFGFBP1 would target miR-9-5p via the predicted site. According to the dual luciferase reporter gene assay, the co-transfection of the miR-9-5p mimic and the circFGFBP1 wild type containing the potential site could significantly decrease the luciferase activity (Figure 4D), suggesting the site was the binding site of circFGFBP1 to sponge miR-9-5p. Moreover, we demonstrated that circFGFBP1 and miR-9-5p were co-located in the cytoplasm (Figure 4E). Together, circFGFBP1 may function as a miRNA sponge for miR-9-5p in human NP cells.

### 2.5. MiR-9-5p Partially Reverses Apoptosis and ECM Degradation Inhibited by circFGFBP1 Upregulation in NP Cells

**Figure 5:**
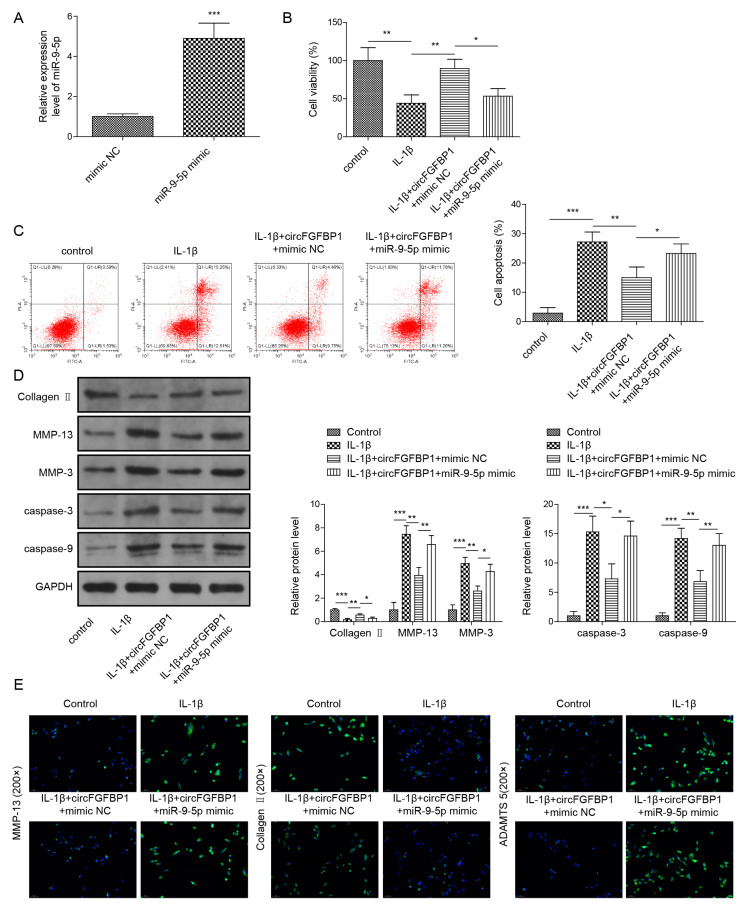
MiR-9-5p reverses apoptosis and ECM degradation inhibited by circFGFBP1 upregulation in NP cells. (**A**) MiR-9-5p expression by qPCR. (**B**) Cell viability by CCK-8 assay. (**C**) Apoptosis in NP cells by flow cytometry. (**D**,**E**) Expressions of ECM proteins by Western blot (**E**) and immunofluorescence (**E**). Data were estimated as mean ± SD. * *p* < 0.05, ** *p* < 0.01, *** *p* < 0.001.

We used a miR-9-5p mimic to increase this miRNA expression in NP cells (Figure 5A). In IL-1β-stimulated cells, miR-9-5p elevation decreased circFGFBP1-increased cell viability (Figure 5B); also, it promoted circFGFBP1-inhibited apoptosis (Figure 5C). Without IL-1β stimulation, NP cells transfected with the miR-9-5p mimic developed the decreased cell viability (Figure S1C). To reverse the role of circFGFBP1 in cells stimulated by IL-1β, miR-9-5p upregulation inhibited Collagen II and Aggrecan and enhanced MMP-3, MMP-13, cleaved Caspase-3, cleaved Caspase-9 and ADAMTS5 (Figure 5D,E). These findings suggested that circFGFBP1 may improve NP cell damage by sponging miR-9-5p.

### 2.6. BMP2 Is Identified as a Target of miR-9-5p

**Figure 6:**
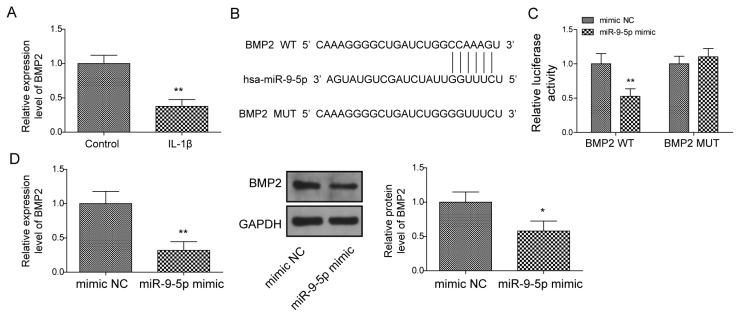
BMP2 is identified as a target of miR-9-5p. (**A**) BMP2 expression under IL-1β stimulation using qPCR. (**B**) The potential site of miR-9-5p located in BMP2 by starBase. (**C**) The relationship between miR-9-5p and BMP2 via dual luciferase reporter gene assay. (**D**) BMP2 expression by qPCR and Western blot. Data were estimated as mean ± SD. * *p* < 0.05, ** *p* < 0.01.

We observed BMP2 was downregulated under IL-1β stimulated in NP cells (Figure 6A) and, intriguingly, we predicted that BMP2 might be the target of miR-9-5p due to the potential binding site located in the 3′UTR of BMP2 (Figure 6B). Thus, we wondered whether miR-9-5p could bind to BMP2 via the site, leading to the reduction of BMP2 in NP cells. We constructed BMP2-wt and BMP2-mut, which were cloned in a pGL4 vector containing the luciferase reporter gene, respectively. We found that the relative activity of luciferase was decreased when BMP2-wt and the miR-9-5p mimic were co-transfected into cells (Figure 6C). Further, miR-9-5p upregulation resulted in the decreased BMP2 in NP cells (Figure 6D), suggesting miR-9-5p could negatively regulate BMP2 expression during IVDD. 

### 2.7. Inhibition of miR-9-5p Suppresses Apoptosis and ECM Degradation by Targeting BMP2 in NP Cells

**Figure 7:**
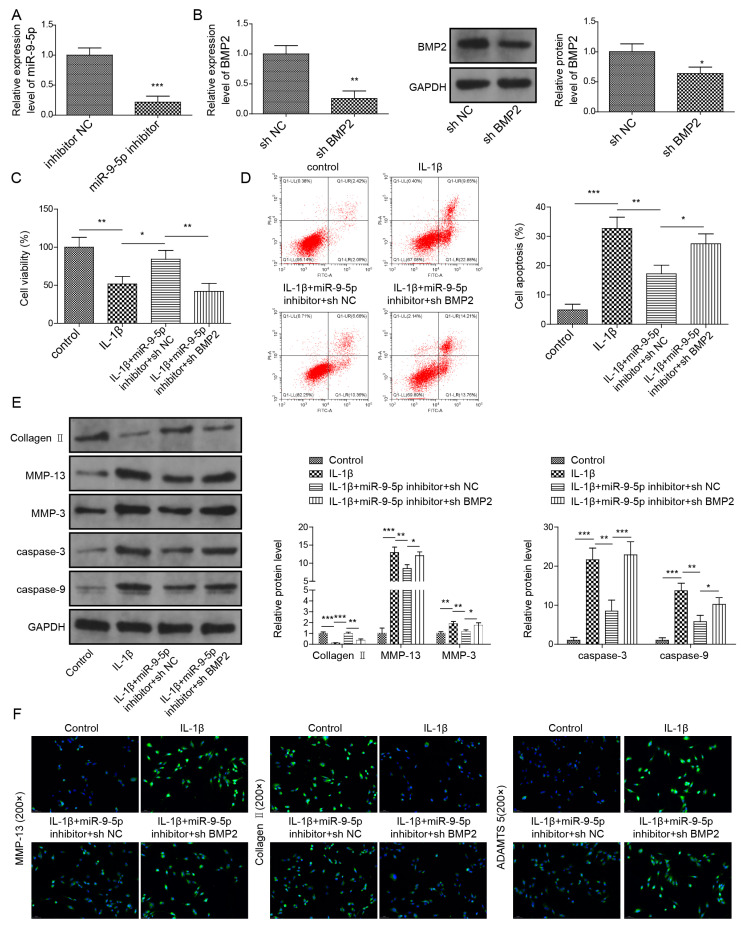
Knockdown of miR-9-5p suppresses apoptosis and ECM degradation by targeting BMP2 in NP cells. (**A**) MiR-9-5p expression by qPCR. (**B**) BMP2 expression by qPCR and Western blot. (**C**) Cell viability by CCK-8 assay. (**D**) Apoptosis in NP cells by flow cytometry. (**E**,**F**) Expressions of ECM proteins by Western blot (**E**) and immunofluorescence (**F**). Data were estimated as mean ± SD. * *p* < 0.05, ** *p* < 0.01, *** *p* < 0.001.

We wondered whether the role of miR-9-5p in NP cells was mediated by BMP2; therefore, we used a miR-9-5p inhibitor and sh-BMP2 to downregulate miR-9-5p (Figure 7A) and BMP2 (Figure 7B) in NP cells, respectively. MiR-9-5p silence elevated the cell viability of NP cells stimulated by IL-1β; nevertheless, BMP2 downregulation made the cell viability decrease to that in IL-1β-stimulated cells (Figure 7C). Additionally, the concomitant downregulation of miR-9-5p and BMP2 elevated miR-9-5p-inhibted apoptosis in IL-1β-stimulated cells (Figure 7D). Owing to miR-9-5p silence, Collagen II and Aggrecan were elevated but MMP-3, MMP-13, cleaved Caspase-3, cleaved Caspase-9 and ADAMTS5 were reduced in IL-1β-stimulated cells (Figure 7E,F). However, these changes were reversed by BMP2 downregulation (Figure 7E,F). Collectively, miR-9-5p confirmed its function in NP cells by targeting BMP2.

### 2.8. Overexpression of circFGFBP1 Alleviates the Progression of IVDD In Vivo

**Figure 8:**
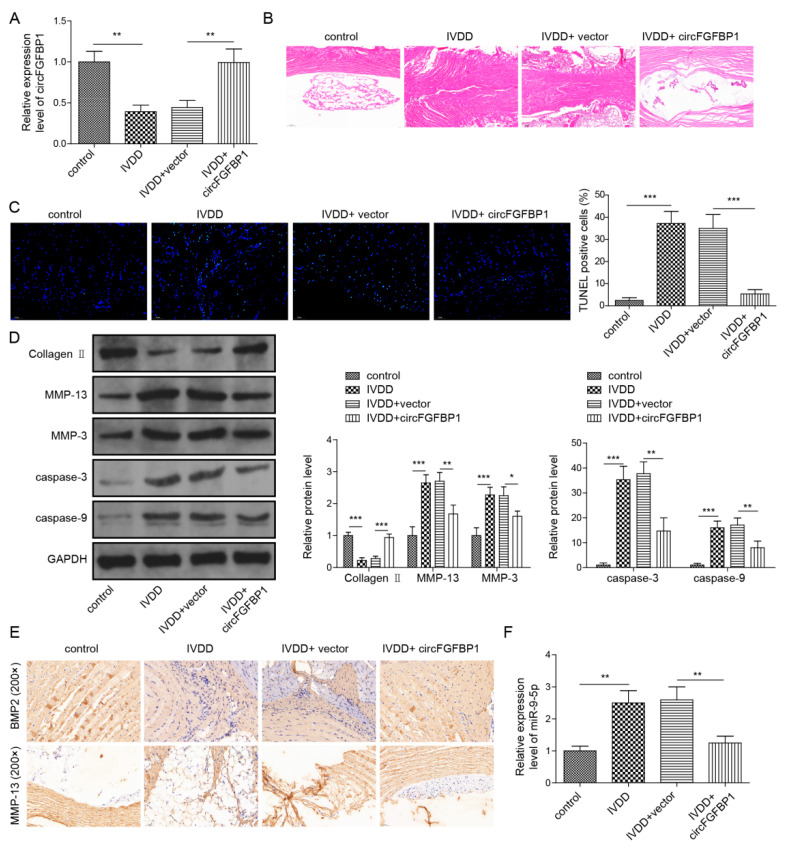
Overexpression of circFGFBP1 alleviates the progression of IVDD in vivo. (**A**) CircFGFBP1 expression in NP tissues by qPCR. (**B**) Histopathological changes by H&E staining. (**C**) Apoptosis in NP tissues by TUNEL staining. (**D**) Expressions of ECM proteins in NP tissues by Western blot. (**E**) Expressions of BMP2 and MMP-13 in in NP tissues by immunohistochemistry. (**F**) Expression of miR-9-5p by qPCR. Data were estimated as mean ± SD. * *p* < 0.05, ** *p* < 0.01, *** *p* < 0.001.

To determine the role of the circFGFBP1/miR-9-5p/BMP2 axis in IVDD progression, we injected a circFGFBP1 overexpression vector packaged by adenovirus into the caudal vein of IVDD rats. IVDD caused a circFGFBP1 level decrease in NP tissues; the circFGFBP1 overexpression vector resulted in a circFGFBP1 expression upregulation (Figure 8A). We observed a collapsing disc structure and infiltrating fibrous tissues in the NP tissues undergoing IVDD (Figure 8B). However, circFGFBP1 upregulation mitigated the impairment in the NP tissues (Figure 8B). IVDD induced the serious apoptosis in rats, whereas circFGFBP1 upregulation inhibited IVDD-induced apoptosis (Figure 8C). Due to IVDD, Collagen II was diminished and MMP-13, MMP-3, cleaved Caspase-3 and cleaved Caspase-9 were elevated in NP tissues of rats (Figure 8D). Intriguingly, circFGFBP1 upregulation could reverse IVDD-caused alterations in these proteins (Figure 8D). We determined expressions of BMP2 and MMP-13 based on immunohistochemistry. Obviously, there was increased MMP-13 and decreased BMP2 in rats with IVDD (Figure 8E). A circFGFBP1 increase partly reversed expressions of BMP2 and MMP-13 in rats with IVDD (Figure 8E). Importantly, we determined that miR-9-5p was increased in rats with IVDD, but circFGFBP1 upregulation significantly reduced its expression during IVDD (Figure 8F). In general, circFGFBP1 could improve IVDD via the miR-9-5p/BMP2 axis.

## 3. Materials and Methods

### 3.1. Collection of Human NP and Serum Samples

We recruited spinal surgery patients admitted to our hospital from January 2019 to December 2020. Degenerative intervertebral disc samples (*n* = 15) were collected from patients with IVDD undergoing discectomy. Normal intervertebral disc samples (*n* = 15) were obtained from patients with traumatic vertebral fracture undergoing decompressive surgery due to neurological deficits. Each patient signed the informed consent for the present study. This clinical study was approved by the ethics committee of our institution (approval number: 2018060). The clinical characteristics of the patients are shown in Table 1. There was no statistical difference in the characteristics of patients between the IVDD and the normal groups (Table 1). The NP cells were isolated from human NP cells by the investigators with unified training. The inclusion criteria of the IVDD patients were: (a) patients who showed the typical clinical symptoms of IVDD; (b) patient with a Pfirrmann’s score ≥ grade 3 (Pfirrmann’s score was used to evaluate the degree of intervertebral disc degeneration). The inclusion criterion of the normal group was: spinal surgery patients with a Pfirrmann’s score < grade 3. The exclusion criteria of the subjects were: (a) patients with abnormal routine blood test, ESR and CRP; (b) patients with an intervertebral disc infection or a spinal tumor using lumbar magnetic resonance. During the operation, the surgeon took out the intervertebral disc tissue of the patients. The intervertebral disc tissue was washed by PBS. Then, the annulus fibrosus and nucleus pulposus were separated. Nucleus pulposus tissues were put into the test tube for numbering and stored at –80 °C. In addition, serum samples from the patients were collected and stored. 

### 3.2. Isolation, Culture and Grouping of Primary NP Cells

Primary NP cells were isolated from the non-degenerative intervertebral disc in patients with a spine fracture according to a previous method [9]. NP tissues were cut into approximate 1 mm^3^ segments and then digested by 0.25% trypsin (0.5 h; Gibco, Grand Island, NY, USA) and type II Collagenase (4 h; Invitrogen, Carlsbad, CA, USA) at 37 °C. Dulbecco’s modified Eagle’s medium (DMEM; Gibco) containing 10% fetal bovine serum (Gibco) was added to end the digestion. After the centrifugation at 1000× *g* for 10 min, cells were resuspended in culture medium. In a 37 °C, 5% CO_2_ incubator, NP cells were cultured with DMEM containing 10% fetal bovine serum and 1% penicillin–streptomycin (Solarbio, Beijing, China). 

The overexpression vectors of FOXO3 and circFGFBP1 were constructed based on the pcDNA3.1 vector (Invitrogen). The downregulation plasmid of BMP2 based on the short hairpin RNA (shRNA) sequence (sh-BMP2) and its negative control (sh-NC) were purchased from RiboBio (Guangzhou, China). A miR-9-5p mimic/inhibitor was constructed to upregulate or downregulate the miR-9-5p expression in cells. NP cells were divided into the following groups: control, IL-1β, IL-1β + vector, IL-1β + FOXO3, IL-1β + circFGFBP1, IL-1β + circFGFBP1+NC mimic, IL-1β + circFGFBP1 + miR-9-5p mimic, IL-1β + miR-9-5p inhibitor + sh-NC and IL-1β + miR-9-5p inhibitor + sh-BMP2. To mimic the cell model of IVDD, IL-1β (Sigma-Aldrich, St. Louis, MO, USA) stimulation was processed in NP cells via a 24 h incubation at the dose of 10 ng/mL. Cell transfection was performed using lipofectamine 3000 (Invitrogen) 48 h before IL-1β stimulation. Cells in the control group were cultured without cell transfection and IL-1β stimulation. 

### 3.3. RNase R Treatment and Actinomycin D Assay

Total RNA (4 μg) isolated from NP cells was incubated with or without 3 U/μg RNase R (Epicenter Technologies, Madison, WI, USA) for 30 min at 37 °C. In addition, we used 10 μmol/L actinomycin D (Cell Signaling Technology, Danvers, MA, USA) to treat primary NP cells for 0, 4, 8, 12 and 24 h, respectively. Then, circFGFBP1 and FGFBP1 mRNA expression levels were examined via quantitative real-time polymerase chain reaction (qPCR).

### 3.4. Fluorescence In Situ Hybridization (FISH)

FITC-labeled circFGFBP1 and miR-9-5p probes were constructed to determine the subcellular location of circFGFBP1 and miR-9-5p. Cells cultured in 48-well plates were fixed in 100% ethanol for 5 min at room temperature and then incubated with 100% Triton X-100 for 15 min at room temperature. Then, cells were incubated with FITC-labeled probes according to the guidelines of the RNA FISH kit (Thermo Fisher Scientific, Waltham, MA, USA). The fluorescence images were observed using a fluorescence microscope (Olympus, Tokyo, Japan). 

### 3.5. Cell Counting Kit 8 (CCK-8) Assay

We used CCK-8 (ab228554, Abcam, Cambridge, MA, USA) to measure the viability of NP cells. After a 24 h culture in a 37 °C, 5% CO_2_ incubator, cells planted in 96-well plates were incubated with a CCK-8 solution at the dose of 10 μL/well and then cultured for 4 h at 37 °C. The absorbance of samples was read at 450 nm using a microplate reader (Thermo Fisher Scientific). 

### 3.6. Flow Cytometry for Apoptosis Measurement

An Annexin V-FITC apoptosis detection kit (Abcam) was used to measure apoptosis in NP cells; 1 × 10^5^ cells were incubated with 5 μL of Annexin V-FITC and 5 μL of propidium iodide for 5 min at room temperature, protected from light. The apoptosis quantification was performed under a flow cytometry (Beckman Coulter, Inc., Miami, FL, USA). 

### 3.7. qPCR Assay

Cell or tissue lysates were incubated with Trizol reagent (Thermo Fisher Scientific) to extract total RNA. Using a one-step reverse-transcription qPCR kit (Tiangen, Beijing, China), RNA was transcribed into cDNA and quantified under a real-time PCR system (Applied Biosystem, Foster City, CA, USA). Primers of circFGFBP1, FGFBP1, FOXO3, miR-9-5p and BMP2 were designed and synthesized by Sangon Biotech (Shanghai, China). We used a nuclear/cytosol fractionation kit (Biovision, 152 Grove Street, Waltham, MA, USA) to collect nuclear and cytoplasmic fractions from NP cells. Lastly, we quantified circFGFBP1 expression in the cytoplasm and nucleus to investigate its distribution of circFGFBP1 in NP cells. GAPDH was used as the internal reference for the cytoplasmic fraction or circRNA/mRNA and U6 was used for the nuclear fraction or miRNA. The relative expression levels of genes were calculated using the 2^-ΔΔCt^ method. The primer sequences are listed in Table 2.

### 3.8. Western Blot

TBST solution: Tris buffered saline (Cell Signaling Technology) containing Tween 20. Blocking buffer: TBST containing 5% *w*/*v* defatted milk powder (Cell Signaling Technology). Cells or tissues were incubated in RIPA lysis buffer (Beyotime, Shanghai, China) to extract proteins, followed by centrifugation at 10,000× *g* for 5 min. The supernatant was used for Western blot. A BCA kit (Beyotime) was used to determine protein concentration before the electrophoresis using SDS-PAGE (Bio-Rad, Hercules, CA, USA). Through a Trans-Blot system (Bio-Rid, Hercules, CA, USA), the protein was transferred to nitrocellulose membranes (Cell Signaling Technology). After washing by TBST solution for 3 times, the membranes were incubated with the blocking buffer for 1 h at room temperature. Membranes were incubated with the primary antibody at 4 °C, followed by the incubation with the secondary antibody (ab288151, 1:10,000, Abcam) at room temperature for 1 h. Membranes were incubated with ECL reagent (Cell Signaling Technology) for 1 min, protected from light. An X-ray image system (Bio-Rid) was used to image and analyze blots on membranes. GAPDH was used for the internal reference. Primary antibodies are listed in Table 3. 

### 3.9. Bioinformatic Prediction for Binding Site

The binding site between FOXO3 and the circFGFBP1 promoter was predicted by the JASPAR database (https://jaspar.genereg.net/ (accessed on 17 March 2023)). To obtain the potential site of transcription factor, the promoter sequence of the encoding gene of circFGFBP1 was used for analysis in the JASPAR database. We used the starBase tool (https://starbase.sysu.edu.cn/ (accessed on 17 March 2023)) to screen and identify the circRNA/miRNA/mRNA interaction work related to circFGFBP1. 

### 3.10. Chromatin Immunoprecipitation (ChIP)

The 4 × 10^6^ cells were incubated with 1% fresh formaldehyde (Sigma-Aldrich, St. Louis, MO, USA) for 10 min at room temperature and with 2 mL glycine solution (Cell Signaling Technology) for 5 min at room temperature. After the centrifugation at 2000× *g* for 5 min at 4 °C, cell pellets were incubated with micrococcal nuclease for 20 min at 37 °C to obtain DNA. After the purification procedure, DNA was incubated with protein A/G beads (Beyotime) binding to anti-FOXO3 (ab70315, 2.5 μg/mg, Abcam) or anti-IgG (ab172730, 0.5 μg, Abcam) overnight at 4 °C. Beads were collected to be washed by low salt immune complex wash buffer (Beyotime), high salt immune complex wash buffer (Beyotime), LiCl immune complex wash buffer (Beyotime) and TE buffer (Beyotime), successively. The enrichment and purification of DNA was performed by a PCR clean up kit (Beyotime). DNA expression was quantified by qPCR. 

### 3.11. Dual Luciferase Reporter Gene Assay

To determine the FOXO3-circFGFBP1 binding axis, base mutation was performed in the promoter of circFGFBP1 according to the binding site. The mutant (circFGFBP1-mut) and the wild type (circFGFBP1-wt) were cloned in a pGL4 vector (Promega, Madison, WI, USA) containing the reporter gene of firefly luciferase, respectively. Each pGL4 vector was co-transfected with pcDNA3.1 vector and pcDNA3.1-FOXO3 in cells, respectively. A total of 48 h after the transfection, luciferase activity was measured using a microplate reader (Thermo Fisher Scientific). The reporter gene of Renilla luciferase was used for the internal reference. To determine the circFGFBP1/miR-9-5p/BMP2 axis, circFGFBP1-wt, circFGFBP1-mut, BMP2-wt and BMP2-mut were constructed to co-transfect with the NC mimic and the miR-9-5p mimic in cells, respectively. The next steps followed the above steps. 

### 3.12. RNA Immunoprecipitation (RIP)

The 1 × 10^7^ cells were resuspended in phosphate-buffered saline (PBS; Thermo Fisher Scientific), followed by centrifugation at 1000× *g* for 5 min. Cell pellets were incubated with a polysome lysis buffer containing a protease inhibitor cocktail (Roche, Basel, Switzerland) and an RNase inhibitor (Promega). Subsequently, cell lysis was incubated with DNase I (Roche) for 10 min at 37 °C, followed by centrifugation at 16,000× *g* for 10 min at 4 °C. The supernatant was incubated with anti-AGO2 (ab186733, 1:50, Abcam) or anti-IgG (ab172730, 0.5 μg, Abcam) overnight at 4 °C and then with protein A/G beads (Beyotime) overnight at 4 °C. The bead complex was collected to perform RNA extraction. CircFGFBP1 and miR-9-5p in RNA extract were quantified by qPCR. 

### 3.13. Immunofluorescence (IF) Assay

Cells cultured in 96-well plates were fixed in 4% formaldehyde for 15 min at room temperature, followed by the blocking of the block buffer consisting of PBS, 5% normal goat serum and 0.3% Triton-X 100 for 1 h. Cells were incubated with a primary antibody overnight at 4 °C and then cultured with a secondary antibody linked to fluorescein (ab150077, 1:200 or ab150113, 1:500; Abcam) for 1–2 h at room temperature in the dark. DAPI (Solarbio) was used for nuclear staining. Expressions of MMP-13 (ab39012, 1:100, Abcam), Aggrecan (sc-70334, 1:50, Santa Cruz Biotechnology, Santa Cruz, CA, USA) and ADMTS5 (ab246975, 1:100, Abcam) were determined by IF assay. 

### 3.14. Animal Experiment

Male Sprague Dawley (SD) rats (*n* = 32) aged 3 months were used to construct the animal model of IVDD. Animals purchased from Vital River (Beijing, China) were housed under 60% humidity with a 12-h cycle of light/dark. They had free access to water and diet. Rats were randomly divided into 4 groups: control, IVDD, IVDD + vector and IVDD + circFGFBP1. Rats in the IVDD group underwent a puncture of a 21-gauge needle at the endplates located in the L4/5 intervertebral disc [14]. The overexpression vector of circFGFBP1 was packaged by adenovirus. Rats in the IVDD + vector and the IVDD + circFGFBP1 groups were injected with the control vector and the circFGFBP1 vector into the caudal vein at the dose of 1 × 10^7^ GC/mL 1 week before IVDD modeling, respectively. The vector injection was performed again at 2 weeks post-modeling. Euthanasia was processed in rats 9 weeks after modeling. The L4/5 intervertebral disc was isolated for investigation. The animal experiments were approved by the ethics committee of our institution (approval number: 2021410). 

### 3.15. Histopathology Observation

We determined the histopathological changes in the frozen sections of the L4/5 intervertebral disc based on an H&E staining kit (Beyotime). Sections were incubated with 4% paraformaldehyde (Beyotime) for 10 min, followed by staining using a hematein reagent for 5 min. Sections were then stained using an eosin reagent for 1 min. After dehydration, transparent sealed sections were observed under a microscope (Olympus). 

We measured expressions of BMP2 and MMP13 in the intervertebral disc using immunohistochemistry. The frozen sections were incubated with 3% formaldehyde for 15 min at room temperature, followed by the incubation with peroxidase for 10 min at room temperature. Antigen repair in sections was processed by microwave heating in a citric acid buffer. Sections were blocked in the block buffer consisting of TBST, 0.3% Triton X-100 and 5% normal goat serum for 1 h at room temperature. Sections were cultured with a diluted primary antibody overnight at 4℃, followed by incubation with a secondary antibody (GTX213110-01, 1:500, GeneTex, Irvine, CA, USA) at 37 °C for 10 min. Sections were labeled by HRP-linked streptavidin for 20 min at 37 °C, followed by the reaction with DAB reagent for 5 min at room temperature. Sections was stained by a hematein reagent for 5 min. After dehydration in ethanol and xylene and incubation with neutral balsam, expressions of BMP2 and MMP-13 were determined using the microscope (Olympus, Tokyo, Japan). Anti-BMP2 (GTX64355, 1:50) and anti-MMP-13 (GTX100665, 1:200) were purchased from GeneTex. 

We used a TUNEL staining kit to detect apoptosis in NP tissues. Sections were fixed in 4% paraformaldehyde for 30 min at room temperature, followed by incubation with PBS containing 0.3% Triton-X 100 for 5 min at room temperature. Sections were blocked in 0.3% H2O2 (in PBS) for 20 min at room temperature to inactivate endogenous catalase. Sections were reacted with the TUNEL working solution (Beyotime) at 37 °C for 1 h. An anti-fluorescence quenching sealing liquid was added in sections. Apoptosis in NP was determined using the fluorescent microscope (Olympus). 

### 3.16. Enzyme-Linked Immunosorbent Assay (ELISA)

The levels of IL-1*β* in the serum samples were detected using a Human IL-1*β* ELISA kit (E-EL-H0149c, Elabscience Biotechnology, Wuhan, China) according to the kit’s instruction.

### 3.17. Statistical Analysis

This study is a double-blind trial. During the study, observers were blinded to the grouping assignment. Independent evaluators for data were blinded to the experiment design. Experiments in the present study were repeated 3 times to ensure data reliability and data were estimated as mean ± standard deviation (SD). GraphPad Prism version 7.0 (GraphPad Software, San Diego, CA, USA) or SPSS version 22.0 (IBM, Armonk, NY, USA) was used for statistical analysis. The Shapiro–Wilk test was used for the normality test. The data in this study followed the normal distribution. Thus, the comparison between the 2 groups was performed by unpaired two-tailed Student’s *t*-test; among multiple groups (≥3) was carried out based on one-way analysis of variance (ANOVA), followed by the post multiple comparison of Tukey’s multiple comparisons test. We determined the significance in statistics when the *p*-value < 0.05 at a 95% confidence interval. We used nQuery software to estimate the sample size in the clinical study and the animal experiment, with α = 0.05 and 1 − *β* = 85%. The statistical analysis was determined by Student’s *t*-test (2 groups) and ANOVA (≥3 groups). 

## 4. Discussion

IVDD is affected by the disorder of metabolism homeostasis and mainly contributes to low back pain [15]. In the present study, we provide a novel mechanism to modulate proliferation, apoptosis and ECM composition in NP cells during IVDD. Briefly, FOXO3 enhanced the transcription of circFGFBP1 to repress miR-9-5p that functioned as the negative regulator of BMP2 expression in NP cells; circFGFBP1-mediated BMP2 upregulation inhibited death and ECM degradation in NP cells to resist the impairment of IVDD. 

Intriguingly, we observed that the circular transcript of circFGFBP1 was more stable than the linear transcript of FGFBP1 in primary NP cells, which indicated that circFGFBP1 might be the more significant role for maintaining the normal process of NP cells. Additionally, we found that circFGFBP1 was mainly expressed in the cytoplasm of these cells. In the animal model of IVDD, we determined that circFGFBP1 was downregulated in NP tissues with fibrotic tissue infiltration. Furthermore, circFGFBP1 expression was reduced in impaired NP cells stimulated by IL-1β. The abovementioned results suggest the potential role of circFGFBP1 to regulate the integrity of NP during IVDD. Based on these findings, we upregulated the circFGFBP1 expression in NP to investigate its role in IVDD. circFGFBP1 upregulation improved the histopathological injury of NP tissues undergoing IVDD and protected NP cells from IL-1β stimulation. Additionally, circFGFBP1 upregulation inhibited ECM degradation and glycosaminoglycan loss during IVDD. circFGFBP1 may be the protective role of NP cells to resist IVDD-related impairment. The NP cell is a chondrocyte-like cell that resists compressive forces on the intervertebral disc [16]. The phenotype of NP cells contributes to maintaining the homeostasis of the intervertebral disc via modulating the composition and structure of ECM. According to our findings, circFGFBP1 can restore the population of NP cells to inhibit the loss of resident cells and rescue the metabolic phenotype, thereby improving the tissue injury due to IVDD [17]. 

We first demonstrated that FOXO3 could enhance the circFGFBP1 expression in NP cells via targeting its promoter. FOXO3 is a transcription factor that modulates the promoter activity of genes related to the lifecycle of cells [18,19]. We found that FOXO3 could promote the transcription of circFGFBP1 via a binding site located in the promoter of circFGFBP1. FOXO3 is found to be downregulated in human degenerated discs [20]. Similarly, we observed that FOXO3 expression was reduced in NP cells undergoing IL-1β stimulation. Possibly, FOXO3 downregulation is the cause of the circFGFBP1 decrease in NP during IVDD. Moreover, FOXO3 has been determined to protect NP cells from nutrient deficiency during IVDD [21]. We found that FOXO3 upregulation reversed apoptosis and ECM degradation in IL-1β-stimulated NP cells. Collectively, FOXO3 enhanced circFGFBP1 expression to induce the regeneration of NP cells, which contributed to restoring the homeostasis of the intervertebral disc. 

We considered the downstream mechanism of circFGFBP1 in impaired NP cells. Significantly, we found that circFGFBP1, as the sponge of miR-9-5p, upregulated BMP2 expression in NP cells to restore the integrity of the intervertebral disc. The circRNA/miRNA/mRNA axis has been determined to be the distinctive role of cellular homeostasis and disease progression [22]. In the initiation and development of IVDD, differentially expressed circRNAs target mRNA stability via sponging miRNA, thereby affecting various cell processes such as adhesion, ECM stiffness and the digestion and absorption of protein [23]. We provide a novel interaction network of circRNA/miRNA/mRNA in IVDD progression. Based on the competing endogenous RNA mechanism, circFGFBP1 can modulate BMP2 to suppress the degenerative histopathological changes in the intervertebral disc and induce the regeneration of NP cells [24,25,26]. Moreover, we also determined the new role of miR-9-5p in NP cells during IVDD. According to our results, miR-9-5p elevation can reverse the protection of circFGFBP1 in NP cells, suggesting that this miRNA is positively associated with the occurrence and development of IVDD. In the models of IVDD, both circFGFBP1 upregulation and miR-9-5p downregulation could repress ECM degradation and the apoptosis in NP cells. miR-9-5p upregulation reversed the changes due to circFGFBP1 overexpression in IVDD, suggesting miR-9-5p was the target of circFGFBP1 in IVDD. 

We found that BMP2 was the target of miR-9-5p in IVDD. We predicted a potential binding site of miR-9-5p located in 3′UTR of BMP2. Then, we performed the site-directed mutation in the possible site in 3′UTR BMP2 (BMP2-mut) and used the complete 3′ UTR of BMP2 without mutation (BMP2-wt) for the control. We cloned BMP2-wt and BMP2-mut in the plasmids containing the luciferase reporter gene, respectively. If miR-9-5p could bind to BMP2 via the site, the luciferase activity in the BMP2-wt group was decreased and that in the BMP2-mut group showed no changes. We transfected the miR-9-5 mimic to easily perceive the change in the luciferase activity due to the binding between miR-9-5p and BMP2. Compared with the NC mimic, the miR-9-5p mimic group showed no change in the luciferase activity of BMP2-mut. Nevertheless, the BMP2-wt group developed the decreased luciferase activity. Thus, we demonstrated that miR-9-5p could directly bind to the site located in BMP2. To validate the role of miR-9-5p in the BMP2 expression, we measured the BMP2 expression in cells transfected with the miR-9-5p mimic. We determined that BMP2 was repressed in NP cells when miR-9-5p was upregulated. 

Furthermore, miR-9-5p may indirectly regulate BMP2 expression. A previous investigation showed that ILF3 increased BMP2 expression via its binding in the promoter of the BMP2 gene [27]. Intriguingly, miR-9-5p has a potential binding site in ILF3, according to starBase analysis. This evidence suggests that miR-9-5p may regulate the ILF3 expression to affect the transcription of BMP2. However, we only verified the direct relationship between miR-9-5p and BMP2. In our findings, the miR-9-5p inhibitor could reverse IL-1β-stimulated changes in proliferation, apoptosis and ECM degradation and, further, BMP2 shRNA could partly offset the reverse role of miR-9-5p under IL-1β stimulation, which suggested that miR-9-5p was capable of inhibiting BMP2 via the binding site and, thereby, it could partly regulate the proliferation, apoptosis and ECM degradation in NP cells. We will reveal the indirect interaction between miR-9-5p and BMP2 in future studies. 

Moreover, we showed FOXO3, circFGFBP1 and miR-9-5p-associated changes in NP cells without IL-1β stimulation. The overexpression of FOXO3 and circFGFBP1significantly enhanced cell viability in NP cells; however, the miR-9-5p mimic inhibited cell viability in NP cells. Based on these results, we can further infer the roles of FOXO3, circFGFBP1 and miR-9-5p in the apoptosis and ECM degradation in NP cells. Possibly, apoptosis and ECM degradation were inhibited with the increased cell viability when FOXO3 and circFBP1 were upregulated in NP cells without IL-1β stimulation. Furthermore, the miR-9-5p mimic promoted apoptosis and ECM degradation when cell viability was suppressed. The changes due to the overexpression of FOXO3, circFGFBP1 or miR-9-5p indicated two hypotheses: (a) overexpression FOXO3 and circFGFBP1 contributes to improve IL-1β-induced injury in NP cells, indicating FOXO3 and circFGFBP1 are the potential targets for IVDD therapy; (b) miR-9-5p aggravates IL-1β-induced cell death and ECM degradation in IVDD, suggesting miR-9-5p expression is positively associated with disease severity of IVDD. Thus, FOXO3, circFGFBP1 and miR-9-5p have potential clinical value in IVDD. 

Based on the above findings, we determined that circFGFBP1 affects ECM degradation and the death in NP cells via the miR-9-5p/BMP2 axis. Given the role of FOXO3 in the circFGFBP1 expression, we finally demonstrated that FOXO3-activated circFGFBP1 inhibits ECM degradation and NP cell death via the miR-9-5p/BMP2 axis in intervertebral disc degeneration in vivo and in vitro. However, the role of miR-9-5p needs to be deeply explored in follow-up studies. 

We provide a novel FOXO3-related circRNA/miRNA/mRNA axis in IVDD. However, there are some limitations as follows: (a) the samples could be increased in further studies to verify our findings in this study on a broader scale; (b) we did not investigate the role of FOXO3, circFGFBP1, miR-9-5p or BMP2 in the diagnosis and prognosis of IVDD. We plan to conduct a 5-year follow-up study with endpoints such as mortality and reoccurrence and to evaluate the potential value of FOXO3/circFGFBP1/miR-9-5p/BMP2 via a receiver operating characteristic analysis and survival curve. Furthermore, the COX analysis is used to determine the risk of FOXO3, circFGFBP1, miR-9-5p and BMP2 in IVDD prognosis. 

## 5. Conclusions

In summary, we provide a novel mechanism of circFGFBP1 in IVDD. At the molecular level, FOXO3 enhances circFGFBP1 expression at the transcription level, which causes BMP2 upregulation due to the sponge of circFGFBP1 on miR-9-5p. BMP2 upregulation inhibits apoptosis and ECM degradation in NP cells during IVDD. Importantly, our findings show a new insight into IVDD treatment based on circFGFBP1-mediated regeneration of NP cells. Our findings suggest that circFGFBP1, FOXO3 and miR-9-5p are the potential targets of IVDD treatment. circFGFBP1 upregulation, FOXO3 overexpression and miR-9-5p silence can promote the therapy of IVDD. Additionally, circFGFBP1, FOXO3 and miR-9-5p are capable of distinguishing IVDD and non-IVDD patients as the biomarkers of IVDD diagnosis.

## Figures and Tables

**Table 1:** The characteristics of the patients.

Variable	Normal	IVDD	*p* Value
Age (years)	35.6 ± 11.2	34.2 ± 10.7	0.778 (a)
Body mass index (kg/m^2^)	23.6 ± 2.4	23.8 ± 2.1	0.845 (a)
Sex (*n*, %)			0.656 (b)
Male	9 (60%)	7 (46.7%)	
Female	6 (40%)	8 (53.3%)	

Data are presented as the mean ± SD or count (%). a, Student’s *t*-test. b, Two-sided chi-squared test.

**Table 2:** The primer sequences used in this study for qPCR.

Name	Primer Sequences (5′-3′)
circFGFBP1	Forward: TGTGTTTGAGCAGCGAAGAG
	Reverse: AGAGCAGGGTGAGGCTACAG
FGFBP1	Forward: TGGCAAACCAGAGGAAGACTGC
	Reverse: GGAACCCGTTCTCTTTTGACCTC
FOXO3	Forward: TCTACGAGTGGATGGTGCGTTG
	Reverse: CTCTTGCCAGTTCCCTCATTCTG
miR-9-5p	Forward: AGCTTGCTGCACCTTAGTCT
	Reverse: TGTGTGCGGCTAGAACATCC
BMP2	Forward: CTATCCCCACGGAGGAGTTT
	Reverse: TGTCCAAAAGTCTGGTCACG
U6	Forward: CTCGCTTCGGCAGCACA
	Reverse: AACGCTTCACGAATTTGCGT
GAPDH	Forward: AAGGTCGGAGTCAACGGATTTG
	Reverse: CCATGGGTGGAATCATATTGGAA

**Table 3:** The primary antibodies used in this study for Western blot.

Name	Manufacturer/Product Number	Dilution
FOXO3	Abcam/ab109629	1:5000
BMP2	Abcam/ab284387	1:1000
Collagen II	Abcam/ab188570	1:2000
MMP-13	Abcam/ab39012	1:3000
MMP-3	Abcam/ab52915	1:4000
cleaved Caspase-3	Cell Signaling Technology/#9661	1:1000
cleaved Caspase-9	Cell Signaling Technology/#20750	1:1000
GAPDH	Abcam/ab9485	1:2500

## Data Availability

The datasets used or analyzed during the current study are available from the corresponding author on reasonable request.

## Supplementary Materials

The following supporting information can be downloaded at: https://www.mdpi.com/article/10.3390/ph16030473/s1, Figure S1: The effect of overexpression of FOXO3, circFGFBP1 and miR-9-5p on NP cell viability. (A–C) The role of overexpression of FOXO3 (A), circFGFBP1 (B) and miR-9-5p (C) in cell viability measured by CCK-8 assay. Data were estimated as mean ± SD. * *p* <0.05, ** *p* < 0.01.
