# Correction: Tan et al. FOXO3-Activated circFGFBP1 Inhibits Extracellular Matrix Degradation and Nucleus Pulposus Cell Death via miR-9-5p/BMP2 Axis in Intervertebral Disc Degeneration In Vivo and In Vitro. *Pharmaceuticals* 2023, *16*, 473

**DOI:** 10.3390/ph18091367

**Published:** 2025-09-12

**Authors:** Yanlin Tan, Xiaobin Wang, Yi Zhang, Zhehao Dai, Jing Li, Chuning Dong, Xingwang Yao, Chang Lu, Fei Chen

**Affiliations:** 1 Department of Nuclear Medicine, The Second Xiangya Hospital of Central South University, Changsha 410011, China; 2 Department of Spine Surgery, The Second Xiangya Hospital of Central South University, No.139, Renmin Middle Road, Changsha 410011, China; 3 Department of Surgery Room, The Second Xiangya Hospital of Central South University, Changsha 410011, China

## Error in Figure

In the original publication [1], there was a mistake in “Knockdown of miR-9-5p suppresses apoptosis and ECM degradation by targeting BMP2 in NP cells. (**A**) MiR-9-5p expression by qPCR. (**B**) BMP2 expression by qPCR and Western blot. (**C**) Cell viability by CCK-8 assay. (**D**) Apoptosis in NP cells by flow cytometry. (**E**,**F**) Expressions of ECM proteins by Western blot (**E**) and immunofluorescence (**F**). Data were estimated as mean ± SD. * *p* < 0.05, ** *p* < 0.01, *** *p* < 0.001” as published, in which the plot of the control group is duplicated from Figure 2D in the work of Wu et al., 2022 [2]. Upon thorough investigation, this error resulted from erroneous provision of image materials by a third-party collaborator. The corrected Figure 7D appears below. With this correction, the plot of the control group in Figure 7D has been replaced with a new plot. All authors apologize for this error. The authors state that the scientific conclusions are unaffected. This correction was approved by the Academic Editor. The original publication has also been updated.

## Figures and Tables

**Figure 7:**
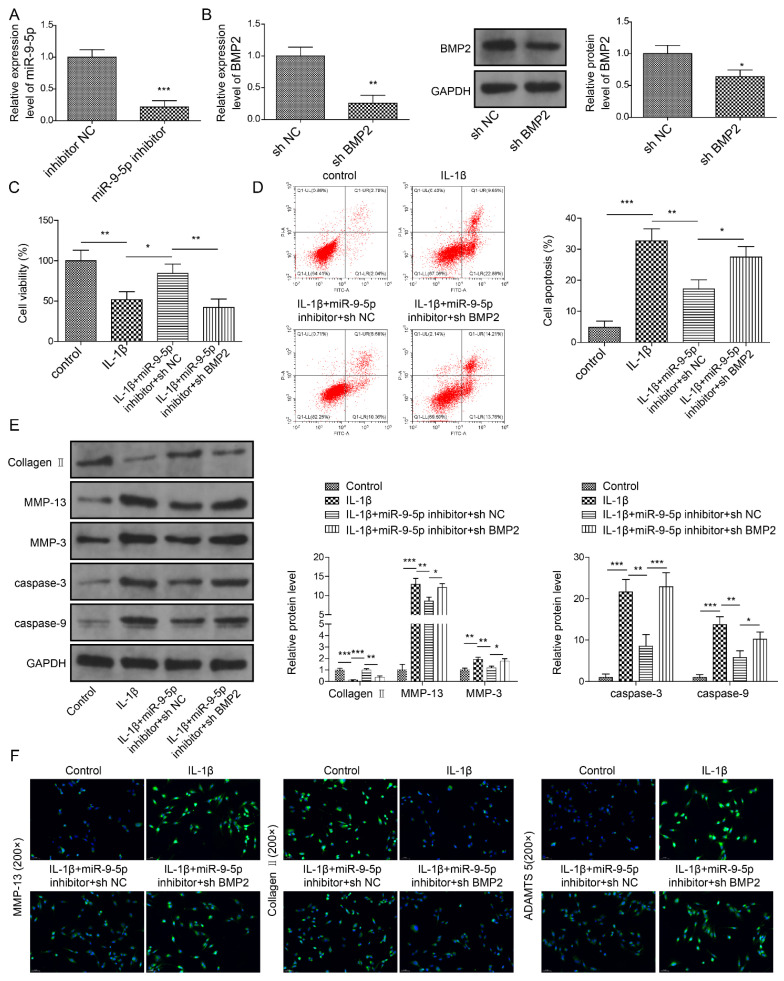
Knockdown of miR-9-5p suppresses apoptosis and ECM degradation by targeting BMP2 in NP cells. (**A**) MiR-9-5p expression by qPCR. (**B**) BMP2 expression by qPCR and Western blot. (**C**) Cell viability by CCK-8 assay. (**D**) Apoptosis in NP cells by flow cytometry. (**E**,**F**) Expressions of ECM proteins and apoptosis-related proteins by Western blot (**E**) and immunofluorescence (**F**). Data were estimated as mean ± SD. * *p* < 0.05, ** *p* < 0.01, *** *p* < 0.001.
