# Supplementary material for: FOXO3-Activated circFGFBP1 Inhibits Extracellular Matrix Degradation and Nucleus Pulposus Cell Death via miR-9-5p/BMP2 Axis in Intervertebral Disc Degeneration In Vivo and In Vitro
Source: Pharmaceuticals (Basel). 2023 Mar 22;16(3):473. doi: 10.3390/ph16030473 (PMC10057604; doi:10.3390/ph16030473)
Supplement: Supplementary file 1 [file pharmaceuticals-16-00473-s001.zip › pharmaceuticals-2220845-supplementary.pdf]

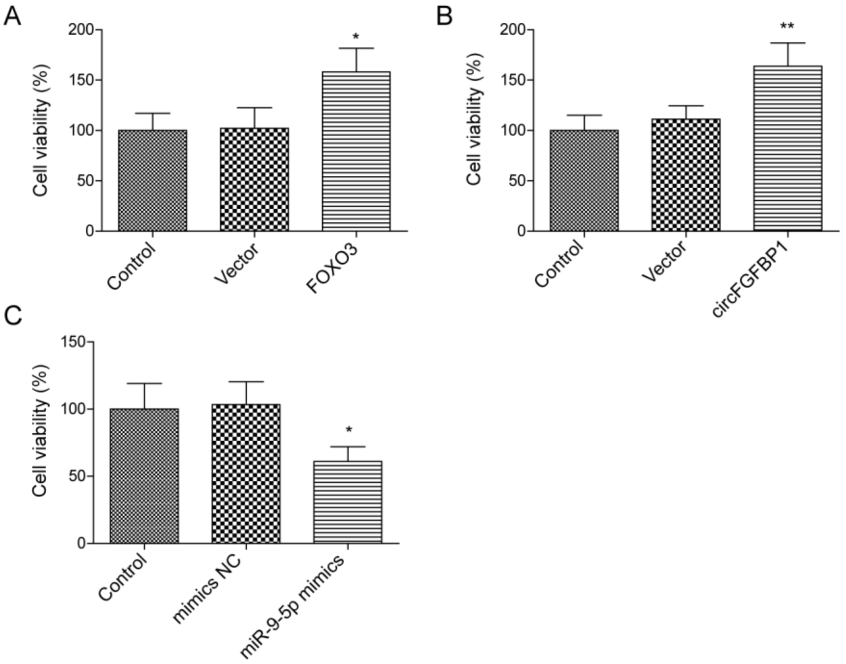

Figure S1. The effect of overexpression of FOXO3, circFGFBP1 and miR-9-5p on NP cell viability. (A-C) The role of overexpression of FOXO3 (A), circFGFBP1 (B) and miR-9-5p (C) in cell viability measured by CCK-8 assay. Data were estimated as mean ± SD. * p <0.05, ** p <0.01.
